# How to optimize tuberculosis case finding: explorations for Indonesia with a health system model

**DOI:** 10.1186/1471-2334-9-87

**Published:** 2009-06-08

**Authors:** Riris A Ahmad, Yodi Mahendradhata, Jane Cunningham, Adi Utarini, Sake J de Vlas

**Affiliations:** 1 Department of Public Health, Faculty of Medicine, Gadjah Mada University, Jogjakarta, Indonesia; 2 Department of Public Health, Erasmus MC, University Medical Center, Rotterdam, The Netherlands; 3 Department of Public Health, Institute of Tropical Medicine, Antwerp, Belgium; 4 WHO/TDR, Geneva, Switzerland

## Abstract

**Background:**

A mathematical model was designed to explore the impact of three strategies for better tuberculosis case finding. Strategies included: (1) reducing the number of tuberculosis patients who do not seek care; (2) reducing diagnostic delay; and (3) engaging non-DOTS providers in the referral of tuberculosis suspects to DOTS services in the Indonesian health system context. The impact of these strategies on tuberculosis mortality and treatment outcome was estimated using a mathematical model of the Indonesian health system.

**Methods:**

The model consists of multiple compartments representing logical movement of a respiratory symptomatic (tuberculosis suspect) through the health system, including patient- and health system delays. Main outputs of the model are tuberculosis death rate and treatment outcome (i.e. full or partial cure). We quantified the model parameters for the Jogjakarta province context, using a two round Delphi survey with five Indonesian tuberculosis experts.

**Results:**

The model validation shows that four critical model outputs (average duration of symptom onset to treatment, detection rate, cure rate, and death rate) were reasonably close to existing available data, erring towards more optimistic outcomes than are actually reported. The model predicted that an intervention to reduce the proportion of tuberculosis patients who never seek care would have the biggest impact on tuberculosis death prevention, while an intervention resulting in more referrals of tuberculosis suspects to DOTS facilities would yield higher cure rates. This finding is similar for situations where the alternative sector is a more important health resource, such as in most other parts of Indonesia.

**Conclusion:**

We used mathematical modeling to explore the impact of Indonesian health system interventions on tuberculosis treatment outcome and deaths. Because detailed data were not available regarding the current Indonesian population, we relied on expert opinion to quantify the parameters. The fact that the model output showed similar results to epidemiological data suggests that the experts had an accurate understanding of this subject, thereby reassuring the quality of our predictions. The model highlighted the potential effectiveness of active case finding of tuberculosis patients with limited access to DOTS facilities in the developing country setting.

## Background

Tuberculosis (TB) is one of the world's leading causes of death and disease. Despite the development and availability of effective treatment for several decades, TB still results in 1.6 million deaths per year [1]. Reducing the burden of global TB disease is a part of broader developmental frameworks such as the Millennium Development Goals (MDG). The MDG clearly state that prevalence and mortality rate of TB should be addressed with a goal towards increase in case detection rate (CDR) and treatment success rate within DOTS (Direct Observed Treatment, Short course) strategy. The importance of intensified case finding was acknowledged by the Second Ad Hoc Committee on the Tuberculosis Epidemic, who recognized that DOTS alone was incapable of reducing morbidity and mortality in TB patients [2].

Indonesia is ranked third in the world for tuberculosis burden [1]. Following the nation-wide introduction of DOTS in 1995, the country's National TB Control Program (NTP) has successfully reached the international targets for case detection (> 70%) and treatment success (> 85%) [1]. However, much work remains to be done to ensure these achievements contribute to the TB-related MDG of cutting the prevalence and death rate in half by 2015. The Stop TB Partnership has recommended a six-point strategy to reach these goals. The strategy components are: (1) quality DOTS expansion and enhancement; (2) addressing TB/HIV, multi-drug resistant TB (MDR-TB), and other challenges; (3) health system strengthening; (4) broader engagement of all care providers; (5) empowering communities; and (6) promoting research [3]. This recommendation addresses the importance of intensified efforts towards case finding. The Indonesian NTP has accordingly adopted this strategy into its 2006–2010 strategic framework [4]. Thus the current challenge for the NTP is determining how to best allocate limited resources across the six-point strategy. Clearly, with limited resources, there is a need to rationally set priorities.

In an effort to determine the most efficacious method of case finding, we developed a mathematical model of the health system through which TB patients seek care. We explored the potential use of this model to optimize TB case finding in the Indonesian health system context, by comparing three possible case finding intervention strategies: (1) reducing the number of TB patients who do not seek care; (2) reducing patients' delay in seeking treatment; and (3) engaging non-DOTS providers in the referral of TB suspects to DOTS services. Applying an innovative method, the parameters in the model are quantified by an adjusted Delphi approach using expert knowledge.

## Methods

### Model structure

**Figure 1:**
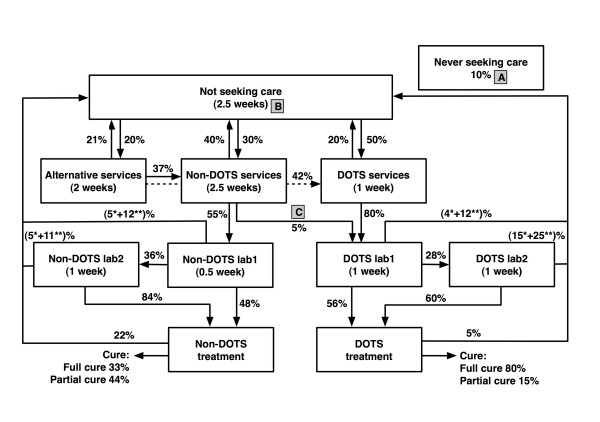
**Basic structure of the health system compartmental model, and corresponding parameters as quantified through expert consultation**. Boxes represent compartments, i.e. stages of the care seeking process. Arrows represent the flow (transition) of individuals between compartments. These individuals are TB suspects/respiratory symptomatics, who are defined as patients with pulmonary TB symptoms serious enough to trigger them to seek health care. TB diagnosis and treatment is not available in the alternative health sector. In DOTS services, the first laboratory examination (*DOTS lab1*) is smear microscopy and the second examination (*DOTS lab2*) is chest X ray, while in Non-DOTS services the situation is vice versa. Parameters are durations and proportions. Durations are defined as the average full duration of being in a certain compartment, thus not including dropout. Proportions concern fractions of TB patients moving into the next compartments. Main outcomes of the model are TB death, cure, and spontaneous recovery. A; B; C are the intervention strategies simulated in this model (see text and Table 3). * Negative test result; ** drop out during diagnostic process. The dotted arrow represents a direct flow from *Alternative *to *DOTS services*.

We developed a compartmental model that describes a TB patients' care seeking behavior and the health system performance of both DOTS and non-DOTS services (e.g. private physicians, nurses, and midwives) in Indonesia. The model was developed with ModelMaker™ version 3.0.3. The model mimics the journey of pulmonary TB patients beginning with the onset of symptoms and follows their care seeking behavior through the health system (Figure 1). Our model population is applicable to all symptomatic TB cases; all smear positive patients, and a subpopulation of smear negative patients with symptoms serious enough to potentially prompt them to seek health care.

There are 11 compartments within the model. Each compartment represents a situation that TB patients encounter during care seeking after the onset of TB clinical symptoms. The model begins with symptomatic TB patients in the population not yet seeking health care *(Not seeking care)*. A proportion of TB patients will never be identified because they never seek help *(Never seeking)*, or prematurely drop out of the health care system before being identified as a TB case. However, the majority of patients will eventually be diagnosed and cured after proper chemotherapy.

TB patients may choose three different types of health care providers:*Alternative, Non-DOTS*, and *DOTS services*; however, adequate (not necessarily standardized) infrastructure for TB diagnosis and treatment can only be accessed through *DOTS *and *Non-DOTS service *providers. The *Alternative services *concern all health providers that do not practice modern western medicine, such as traditional healers and spiritualist. *Alternative services *also include self-medication, both using traditional or modern (over the counter) medicine. We assume that patients can move directly from the *Alternative services *to either *DOTS *or *Non-DOTS services*, while patients in *DOTS *or *Non-DOTS services *need to move back to *Not seeking care *before possibly moving to another sector. If patients do not drop out, they will eventually be identified as TB suspects and referred for diagnostic testing *(DOTS lab1 *or *Non-DOTS lab1)*.

Several potential outcomes may result from the diagnostic process. Following diagnostic testing, patients may be (i) identified as TB positive (i.e. true positive diagnosis) and referred for treatment *(DOTS treatment *and *Non-DOTS treatment)*, (ii) identified as TB negative (i.e. false negative diagnosis) but require additional testing, or (iii) identified as TB negative with a move back to the *Not seeking care *compartment, i.e. drop-out. TB patients who are referred for a second diagnostic test *(DOTS lab2 *and *Non-DOTS lab2) *may follow one of the following courses: (i) diagnosed as TB positive and recommended for treatment, (ii) diagnosed as TB negative and exit the health system, or (iii) fail to complete testing and drop out of the system.

The two final compartments, *DOTS treatment *and *Non-DOTS treatment *are stages where TB patients receive treatment. Some of the referred patients will not receive treatment and drop out of the system. However most patients will be cured or partially cured. Partially cured means TB patients have received treatment for some period of time, but have failed to complete the full course of therapy; thus they are at risk of relapse.

Durations and proportions are parameters of the model that may vary based on strategic intervention or changes in patient behavior. Durations are defined as the average full duration of being in certain compartment; these values do not include the duration of patients who drop out from the compartment. Proportions concern the fractions of TB patients moving into the next compartments. All durations are reported in weeks. The duration and proportions per compartment were translated into transition rates (i.e. number/week) of TB patients moving to adjacent compartments.

Other parameters included in the model are TB death rate and spontaneous recovery rate for TB patients not receiving treatment. Using the Berg study data from the pre-treatment era [5], we estimated that the TB death and recovery rate are approximately 0.3 and 0.2 per year, respectively. These rates correspond to a cumulative TB death and recovery of 40% and 29% after two years, or 73% and 26% after 10 years, respectively, in the absence of effective treatment.

The main outcomes of the model are death, spontaneous cure, and partial- or full cure after treatment. Additional outcomes are the average duration of the care seeking process i.e. duration before treatment, the case detection and cure rate, and TB death rate.

### Parameter quantification process

Parameter quantification was conducted through a two round modified Delphi survey. Due to the poor response rate when using questionnaires, as suggested in the standard Delphi guideline, we instead conducted direct interviews with all participants. Ten experts were identified, and five of these agreed to participate. The experts were selected based on their experience related to the Indonesian TB control program and/or patients care seeking behavior. We made appointments to meet the experts individually for each round. During each individual encounter we posed several questions displayed as a Word file on a laptop computer. Responses were entered directly into the file. We reviewed the answers with the expert before we saved the file as their final response for that round.

During the initial round, participants were first asked to give their view on the basic assumptions of the model structure, in particular the assumptions that some of the patients never seek care, that drop-outs move back to the *Not seeking care *compartment, and the specific diagnostic process sequence. Thereafter they were asked to provide initial estimates for all durations and proportions in the model. In the second round, the mean, median, and extreme responses were presented and participants were invited to revise their estimates given the initial round results. For our analysis we used the reported median of proportions and durations from the second round.

The experts were asked to consider the context of Jogjakarta province, which is located in the central part of Java Island. The province consists of a mix of urban, semi urban, and rural districts. It has a relatively well-established TB control program according to Indonesian standards. It has a TB incidence of 63/100,000, which is the lowest incidence rate in Indonesia [6]. Furthermore, several new TB control interventions, such as public-private hospital involvement, have been piloted in this province with positive outcomes [7].

DOTS services in Indonesia are mainly based in public health centers. However, current NTP policy is to involve hospitals (both public and private) as part of the "engaging all care providers" strategy. In Jogjakarta province, the hospital DOTS linkage had been implemented for five years and reached its maturity prior to conduction of this study. Thus, we can safely assume that the diagnosis procedures and treatment in DOTS hospitals are to a large extent identical to the health centers.

### Model explorations and data validation

Initially, we ran the model to validate the quantifications derived from the experts and verify that they resulted in meaningful predictions. To this end, we used the model to simulate a cohort of symptomatic TB patients and predict in which compartments they would ultimately be located. The following four typical TB program indicators were compared with actual data: duration before treatment, case detection rate, cure rate, and TB death rate. Duration before treatment is the average duration of time from initial location within the *Not seeking care *compartment to the point before entering the *DOTS treatment *compartment for all symptomatic TB cases ultimately ending in the *DOTS treatment *compartment. Case detection is the proportion of a full cohort of symptomatic TB cases starting in *Not *or *Never seeking care *that eventually move to *DOTS treatment *via *DOTS lab1*. Cure rate is the proportion of all symptomatic TB cases in the *DOTS treatment *compartment that are ultimately cured. TB death rate is the proportion of a full cohort of symptomatic TB cases starting in *Not *or *Never seeking care *that die over time due to TB.

For model validation, we compared these four typical TB program indicators with actual data from the national or provincial TB program.

• The duration before treatment was derived from the latest Indonesian TB prevalence survey in 2004 [6].

• In the model, case detection rate was defined as all TB patients (smear positive and smear negative) detected in both DOTS and Non-DOTS services. However, since the existing information available from the Indonesian TB program only includes smear positive TB cases detected in DOTS services, we compared the smear positives detected in *DOTS lab1 *in our model output with the proportion of smear positive detected within the WHO estimated number of total TB cases and the proportion of smear positive detected within the WHO estimated number of smear positive cases. If we assume that symptomatic cases consist of all smear positive cases and between 10%–40% of smear negative TB cases, using the WHO estimated incidence rate of all cases (239/100,000) and the estimated smear positive incidence (108/100,000) for Indonesia [8], the estimated incidence for all symptomatic cases is between 121 and 160 per 100,000 population. Using the estimated smear positive incidence for Jogjakarta province (63/100,000) [6], this translates to an estimated incidence of all symptomatic cases in this province of 71–94/100,000 population. Given the population size of Jogjakarta of 3.44 million in 2005 [9], this leads to 2,400–3,200 symptomatic cases in Jogjakarta. Using the notified number of 1,240 smear positive TB cases reported in Jogjakarta province in 2005 [9], this leads to 39% to 52% of all symptomatic TB cases detected in the DOTS services in Jogjakarta.

• The cure rate is the proportion of new smear positive TB patients cured after treatment received within DOTS services. The reported number of patients cured in the Jogjakarta province TB program surveillance system was 1053 of a total of 1300 TB patients treated in DOTS facilities in Jogjakarta province in 2005 [9]. This represents a cure rate of 81%.

• TB observed death rate is estimated from the 2005 WHO-reported TB mortality rate of Indonesia, 41/100,000 [8]. We adjusted this value to exclude extra-pulmonary TB cases by assuming that the pulmonary TB death rate is 80% of the WHO figure. Furthermore, we assume that the Jogjakarta figure is 60% of the national pulmonary TB death. This lower death rate is based on the general health indicators of Jogjakarta province, which are better than those of Indonesia as a whole. Although the crude mortality rate is slightly higher (7.8 compared to 6.6/100,000), the life expectancy at birth and maternal mortality rate are considerably better in Jogjakarta. Moreover, the health system performance in Jogjakarta is far better than in Indonesia in general; TB is one of the top ten causes of death nationally, while this was not the case in Jogjakarta province. Based on these calculations, the pulmonary TB death rate in Jogjakarta province should be 20/100,000. Given the population size of 3.44 million, we arrive at approximately 700 pulmonary TB deaths in Jogjakarta per year. This means that the 'observed' TB death is between 22%–29% in 2005.

These model outcomes were used as the baseline situation to be compared with three strategies to improve TB case finding. The strategies are as follows: strategy (A) is to reduce the proportion of TB patients who never seek care (*Never seeking care *compartment) by 50%; the second strategy (B) is to reduce patients' delay (reduce duration in *Not seeking care *compartment by 50%); the third strategy (C) is to refer all TB suspect patients from non-DOTS services to DOTS services (i.e. from *Non-DOTS services *to *DOTS lab1 *compartment).

We also considered a different scenario in which a greater proportion of TB patients remain in the *Alternative services *sector. In this model, we assumed that 20% of the TB patients that would have gone to the *DOTS *and *Non*-*DOTS *compartment now move to the *Alternative services *compartment. Also in this model, patients cannot move directly from the Alternative sector to other sectors, but first need to return to the *Not seeking care *compartment. This scenario reflects the health system situation of Indonesian settings outside Java-Bali, where the number of health care facilities (both public and private) is less, and TB patients face greater geographical challenges.

### Sensitivity analysis

A univariate sensitivity analysis was performed to assess how the main results (proportions death and partially cured) change with different model assumptions. To this end we multiplied the baseline values of the transition rates of patients going out of each compartment by 2/3 and 3/2, one rate at a time, and ran the model again. The change in outcomes gives an indication of the sensitivity of the model to the assumed parameter values. By varying transition rates, rather than the expert-derived proportions, all proportions moving out of particular compartments still add up to 100%.

## Results

### Parameter quantification

All experts (Table 1) agreed that the model structure and diagnostic process sequence provided an adequate representation of reality for TB patients and the TB control program in Indonesia. The medians of the expert derived average durations and proportions are reported in Figure 1. In general, the experts suggested that most of the TB patients (90%) seek treatment, with an average duration of 2.5 weeks after their first symptoms before entering the healthcare system. Fifty percent of those seeking treatment go to *DOTS services *and 20% to *Alternative services *as their first health service preference. The experts also acknowledged that *DOTS services *have a better capacity to diagnose and treat TB patients, with a lower drop out rate during treatment and a higher cure rate, compared to *non-DOTS services*. The experts reported a slightly longer duration until treatment for the Non-DOTS sector compared to the DOTS sector. In particular, the assumed duration prior to TB suspicion was 1.5 weeks longer, whereas this was only partly counterbalanced by a 0.5-week shorter duration until diagnosis by the first lab. Furthermore, there were no considerable differences in the experts' estimated parameter values (i.e. durations and proportions) between the first and second Delphi round (data not shown).

**Table 1:** Characteristics of the expert panel

Expert 1:	TB project manager with a vast experience in developing public-private mix among hospitals (TB Hospital DOTS Linkage/HDL) in Jogjakarta province since 2000–2005.
Expert 2:	Microbiologist who works at the university and as laboratory technical consultant for the TB control program. The expert is also involved in the laboratory capacity strengthening, as a part of the TB Hospital DOTS Linkage project in Jogjakarta. Member of national TB laboratory working group. Has recent experience with the study of TB patients' care seeking behavior in this province.
Expert 3:	Urban district TB control program manager with recent experience of conducting a TB patients care seeking behavior study in Jogjakarta province.
Expert 4:	Former rural district TB-control program manager who has health promotion expertise and vast experience in health seeking behavior interventions.
Expert 5:	Medical doctor who was head of a public health center in Jogjakarta during the daytime and a private practitioner in the evening. As the head of the health center the expert has experience in involving private practitioners in the health center catchment area.

### Model simulation

**Figure 2:**
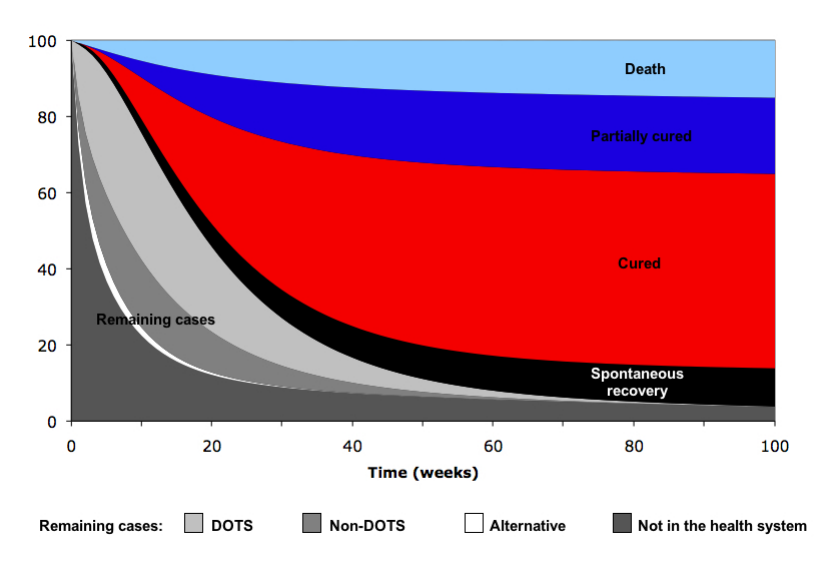
**Cumulative outcome of the care seeking process of TB patients' cohort, starting in *Not seeking care *(90%) and *Never seeking care *(10%) compartments**.

Figure 2 shows a baseline cohort of TB patients during their care seeking process. Most of the patients are cured after treatment. Only a small fraction of TB patients, mainly those who never seek treatment, remain as a source of infection after 100 weeks (approximately 2 years). The model also demonstrates that only a negligible proportion of TB patients stay in the *Alternative services *sector, compared to the *DOTS *or *Non-DOTS services*.

It is reassuring that the Delphi survey quantifications correspond with an average total duration before treatment of 9.3 weeks. This is in agreement with findings from a recent Indonesia national TB prevalence survey, which reported a mean of 10.3 weeks (Table 2). Other critical model outputs are also similar to the existing data. The model prediction for the proportion of smear positive TB patients through DOTS services is within the range of the existing TB case finding data, and the predicted cure rate is slightly less than that found in the data. However, the predicted mortality rate in the model is lower than the estimated range of TB death in Jogjakarta province. As a whole, all model predictions are reasonably close to the reported data, but to some degree overly optimistic.

**Table 2:** Comparison of model output with available data

	Existing data	Model output
Mean duration from the first TB symptoms to treatment (weeks)	10.3^a^	9.3
Proportion (%) of all TB-cases detected as smear positive through *DOTS services*	39 – 52^b^	48.0
Proportion (%) of TB patients in the DOTS services eventually cured	81.0^c^	72.3
Proportion (%) of all TB cases that eventually die	22 – 29^d^	16.6

Model output and data concern symptomatic pulmonary TB cases; both smear positive and part of smear negatives, with symptoms serious enough to potentially prompt them to seek health care.

^a ^based on an Indonesian TB prevalence survey in 2004 [7].

^b ^based on smear positive cases detected in Jogjakarta province TB program, assuming that all smear positive and between 10%–40% smear negative TB cases are symptomatic.

^c ^the reported cure rate from Jogjakarta provincial TB program in 2005 [9].

^d ^based on the WHO estimated death rate for Indonesia as a whole, after correction for 20% extra-pulmonary TB and a 40% lower death rate in Jogjakarta Province [1].

If we consider an alternative situation in which 20% of patients move to the alternative sector, instead of to the *DOTS *and *Non-DOTS services*, the average total duration before treatment would be 12 weeks (not shown). Also for this scenario, most of the TB patients are cured; however a larger proportion of TB cases would die and the cure rate would be lower than in the previous situation (Table 3).

**Table 3:** Predicted effect of three interventions (A, B, C) on a cohort of TB patients (%)

			With intervention		
			
Scenario	Model outcome	No intervention	A	B	C
Baseline^a^	Death	16.6	14.2	15.5	16.5
	Partially cured	19.6	20.7	20.1	11.5
	Cured	52.8	55.7	54.1	61.0
	Spontaneous recovery	11.1	9.5	10.3	11.0
					
More important alternative health sector^b^	Death	18.1	15.8	16.6	18.0
	Partially cured	18.5	19.6	19.2	11.1
	Cured	51.2	54.1	53.1	58.9
	Spontaneous recovery	12.1	10.5	11.1	12.0

A: strategy to reduce the proportion of TB patients who never seek care (*Never seeking *compartment) by 50%;

B: strategy to reduce patients' delay (i.e. reduce duration in *Not seeking care *compartment) by 50%;

C: strategy to refer all TB suspect patients from non-DOTS services to DOTS services (from *Non-DOTS services *to *DOTS lab1 *compartment).

^a^: Simulation using experts estimated parameters

^b^: Simulation using adjusted parameters in the alternative health sector, i.e. if 20% of the flow of TB patients to the *DOTS *and *Non-DOTS services *compartments now move to the *Alternative services *compartment (i.e. the proportion of TB patients move from *Not seeking care *compartment to *Alternative, Non-DOTS, and DOTS services *compartments are 36%, 24%, and 40% respectively), and patients cannot go directly to the *DOTS *and *Non-DOTS services*.

Table 3 illustrates the outcomes of different possible case finding strategies simulated in the model. Strategy A, which is to reduce the number of TB patients who never seek care, is more effective in preventing deaths than strategy B and C. However, strategy C, involving private providers in the referral of TB patients to DOTS facility, leads to a substantially higher proportion of fully cured patients among those receiving treatment. These findings are similar for the scenario with a higher rate of patients initially entering the alternative health sector.

**Figure 3:**
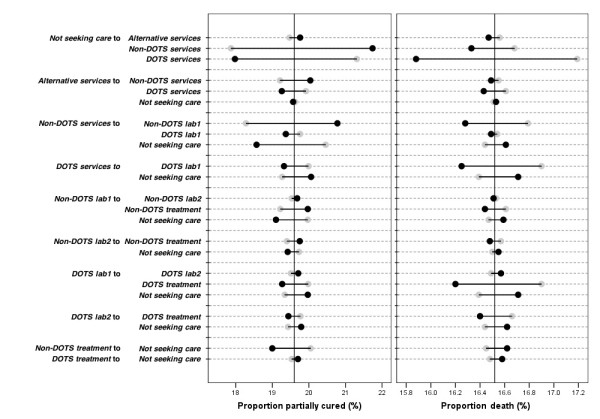
**Sensitivity analysis: impact of changes of the transition rates between the different model compartments on the predicted percentage of partially cured and TB-related death**. Grey dots represent the results using a transition rate of 2/3 times the baseline value, and black dots represent the results using a transition rate of 3/2 times the baseline value. The two vertical black lines represent the respective baseline outcomes.

Figure 3 shows the results of sensitivity analysis. It is clear that changing the model parameters has only a limited impact on outcomes. TB death varied maximally from 15.9 to 17.2 (baseline value of 16.6), while the proportion partially cured varied from 17.9 to 21.7 (baseline value of 19.6). The transition rate from *Not seeking care *to *Non-DOTS services *has the strongest influence on the proportion partially cured. A higher flow to *Non-DOTS services *leads to more patients partially cured, and the proportion TB death slightly decreases as well. The flow from *Not seeking care *to *DOTS services *has by far the biggest influence on TB death, and illustrates that assuming a higher rate of patients going to the *DOTS services *corresponds with a decreased death rate. The proportion partially cured decreases to a comparable degree. Dropout rates (i.e. moving back to *Not seeking care*) show a modest but consistent pattern: a higher rate of dropping out from the non-DOTS compartments of the model always leads to more TB death and less partially cured, whereas dropping out from the DOTS compartments always leads to both more TB death and more partially cured.

## Discussion

This mathematical model guided by parameter values based on expert opinion is a novel approach to study the role of health systems in TB control. Findings from the model simulation suggest that some critical outputs of the model are reasonably close to the existing data, although they tended to be somewhat optimistic. Model explorations show that increasing care-seeking behavior may have a considerable impact on preventing TB death. Additionally, referral of suspected TB cases to DOTS facilities will increase treatment success and prevent partial cure. This finding is similar to a situation with more patients initially entering the alternative health sector. Furthermore, sensitivity analysis revealed that the model predictions are not very sensitive to the assumed parameter values, with the health care seeking rates having the biggest impact.

As the TB control program relies on passive case finding, success is determined by patients' care seeking behavior and health system performance. Previous studies examining TB case finding mainly focus on either the care-seeking behavior of TB patients [10-14] or the health system determinants [7,15-17]. In the 1970s, Piot developed a model of the TB treatment-seeking process, which included both patient- and health system determinants [18]. Two recent studies [19,20] have addressed the interaction between TB patients and health system through modeling; however these studies focused more on the diagnostic process rather than case finding strategies. Our study contributes to the body of knowledge by combining all factors related to TB control into one coherent framework using mathematical modeling. This model takes into account the various types of existing health services, the duration and pattern of care seeking behavior, and TB patient outcome (i.e. death, partial/full cure, and spontaneous recovery).

Our model does not account for the complexities present in the natural history of TB infection, but considers a simple constant risk of death or spontaneous recovery while being in the health system. Including progression and regression of the disease through different stages of severity would allow prediction of the impact of early case detection. Relapse after treatment failure or incomplete cure, MDR-TB, and HIV co-infection will have a different impact on the progression and risk of death in individual TB cases. A more complex model has been designed that considers these factors [21]. Our model also does not include TB transmission. In reality, earlier and better treatment of TB patients will to some extent lower the transmission rate and thereby result in fewer secondary cases in the population. Our predictions on prevented TB deaths are therefore underestimations.

It is difficult to estimate the number of TB patients existing outside government run health services in Indonesia and other developing countries. Consensus methods, such as Delphi, are likely the only means to obtain quantitative information [22,23]. Despite the fact that most of the Delphi studies are not designed to be statistically significant and the ideal panel size has not been identified [22], most studies involve large numbers of participants [22-26]. Therefore, our expert estimated parameters should be interpreted with caution. Still, our results show that the critical outcomes of our model are reasonably close to the existing data, which supports the validity of the parameters (Table 2). It is notable that outcomes of our model are consistently – but to different degrees – more optimistic than epidemiological data. We realize that we could not do a formal statistical validation of the model, since the 'observed' data themselves are partly based on model predictions (by WHO) and additional assumptions.

Because health system characteristics and TB patients' care seeking behavior differs in different regions, our study results may not be directly generalizable to other settings. However, the model can be adapted to others settings provided parameter values are regionally specific.

At present, there is a recognized global urgency to improve TB case detection [27]. The Second Ad Hoc Committee on the Tuberculosis Epidemic also acknowledged that a high DOTS treatment rate alone was incapable of reducing morbidity and mortality, without additional intensified case finding [2]. Heller et al. used modeling simulation and suggested a similar result [28]. There are several different strategies for increasing case finding, such as active case finding (ACF), enhanced case finding (ECF) [29], and expanded passive case finding via increased interaction with private sectors [30]. The difference between ACF and ECF is the level of direct interaction with the target population. ACF is often more labor intensive, involving face-to-face contact and immediate onsite evaluation. ECF educates the population about the symptoms of TB through publicity and education, and encourages self-presentation to medical services [29]. Studies also show that implementation strategies of ACF (radiography screening or symptom based screening [31], tuberculin screening [32], and contact tracing [33-35]), ECF (health education, community involvement [29], or outreach program [36]), and case finding in the public and private sector outside of DOTS program (via involvement of private practitioners in suspected TB case referral [37], diagnosis, and treatment [30,38]) can be feasible and effectively improve TB case finding.

Three strategies are hypothesized to potentially improve TB detection and cure rates. Strategy A can be implemented through symptom-based household contact screening as conducted in Northern Lima, Peru [35]. An example of intervention to achieve strategy B is the community outreach program in rural Southern Ethiopia [36], where a TB campaign was conducted prior to a monthly outreach visit to communities. Strategy C is exemplified by the involvement of private providers, such as informal village doctors in Bangladesh, to refer TB suspects to DOTS facilities [37]. All these interventions lead to substantial improvement in TB case finding. However, the feasibility of actually adopting the suggested intervention strategies in Jogjakarta province remains to be seen. For example, to refer all TB suspect patients to DOTS facilities (strategy C) requires involvement of all private providers. In reality, this may be impractical and difficult to achieve. The feasibility and acceptability of an intervention is to a large extent setting-specific. Any intervention program should be assessed after consultation with local experts. Still, explorations with the model can at least demonstrate the most promising control options with regard to their expected effectiveness.

Effectiveness of a disease intervention is commonly measured by averted disease burden in terms of disability adjusted life years (DALY). In TB, 86% of healthy life lost can be attributed to premature death, while only 14% is due to illness [30]. The model shows that reducing the number of never seeking care TB patients will have the greatest impact on mortality, and will thus result in the greatest improvement in DALY. Further, Borgdorff et al. suggested that ACF would only have limited impact on mortality if the intervention detects patients who would otherwise have been detected through self-reporting [39]. Thus challenges remain in targeting specific high-risk TB sub-groups, e.g. prison inmates, HIV patients, and urban slums dwellers, who have limited access to health services and are less likely to seek care on their own.

The potential effectiveness of implementation strategies should also be judged against the potential costs for implementing such strategies. This is a particularly important consideration in resource-constrained settings such as Indonesia. In these settings, cheaper, albeit less effective, interventions may be a more pragmatic option. Several economic evaluation studies have assessed the cost effectiveness of different TB control strategies, mainly related to DOTS. However, only a limited number of economic evaluations on case finding strategies are available, mainly in the setting of developed countries [30,32,40,41]. Further, no economic evaluation has been published on community based case finding or interventions to refer TB suspects from private providers to DOTS facilities. A study in Dar es Salaam suggests that an active case finding strategy may cost less than $276 US per case treated (1996 exchange rate), in a situation where household cost is the main component of cost for tuberculosis management [42]. The Indonesia NTP's marginal costs of a case successfully treated for 2004–2005 is approximately $450 US [1,43]. This suggests that the ACF strategy in high-risk populations with limited access to DOTS facilities could potentially be considered cost-effective in the Indonesian context. Notwithstanding, formal cost effectiveness analysis comparing ACF, community based case finding, and intervention to refer TB suspects from private providers to DOTS facilities (which is beyond the scope of this study) clearly needs to be carried out before ACF can be advocated as a priority policy option. Results of an economic study of an intervention to refer TB suspects from private providers to DOTS facilities in Jogjakarta province is undergoing evaluation at the time of writing and is expected to shed more light on this issue.

Our model development process has demonstrated significant gaps in knowledge. In general there is little data about the TB diagnosis and treatment process and the outcome in non-DOTS services. Also, little is known about TB patients who never seek care and TB patients who enter the alternative services sector. Another knowledge gap exists regarding the sensitivity of health services in identifying TB suspects. Further, there are limited evidences of cost effectiveness of TB case finding implementation strategies. Thus, further studies are needed to provide such evidence.

## Conclusion

We used mathematical modeling to explore the impact of Indonesian health system interventions on tuberculosis treatment outcome and deaths. We relied on expert opinion to quantify the parameters. The fact that the model output showed similar results to epidemiological data suggests that the experts had an accurate understanding of this subject, thereby reassuring the quality of our predictions. The model highlighted the potential effectiveness of active case finding of tuberculosis patients with limited access to DOTS facilities in the developing country setting.

## Competing interests

The authors declare that they have no competing interests.

## Authors' contributions

RAA contributed to conception and design of the study, acquisition of data, analysis and interpretation of data, drafting and substantially revising the paper. YM contributed to conception and design of the study, acquisition of data, and substantially revising the paper. JC contributed to the conception of the study and substantially revising the paper. AU contributed to design of the study, and substantially revising the paper. SJdV contributed to conception and design of the study, analysis and interpretation of data, and substantially revising the paper. All authors read and approve the final manuscript.

## Pre-publication history

The pre-publication history for this paper can be accessed here:

http://www.biomedcentral.com/1471-2334/9/87/prepub

## References

[B1] WHO Global TB Control: Surveillance, Planning, Financing 2008 Geneva: WHO

[B2] WHO Report of the Meeting of the Second Ad Hoc Committee on the TB Epidemic: Recommendations to Stop TB 2004 Geneva: WHO 15581193

[B3] WHO The Global Plan to Stop TB 2006–2015: Action for Life Toward a World Free of Tuberculosis 2006 Geneva: WHO

[B4] Utarini A Basri C Nadia S Heitkamp P Voskens J Trisnantoro L Ahmad RA Harbianto D Framework of the Indonesian Strategic Plan for Tuberculosis Control: 2006–2010 2006 Jakarta: Ministry Of Health, Republic Of Indonesia

[B5] Murray C Styblo K Rouillon A Jamison DT, et al Tuberculosis Disease Control Priorities in Developing Countries 1993 New York: Oxford University Press

[B6] Soemantri S Lolong DS Senewe FE Wiryawan Y Tejayanti T Pangaribuan L Tjandrarini DH Andayasary L Tuberculosis Prevalence Survey in Indonesia 2004 2005 National Institute of Health Research and Development Ministry of Health – Republic of Indonesia. Jakarta

[B7] Irawati SR Basri C Arias MS Prihatini S Rintiswati N Voskens J Kimerling ME Hospital DOTS linkage in Indonesia: a model for DOTS expansion into government and private hospitals Int J Tuberc Lung Dis 2007 11 33 39 17217127

[B8] WHO Global TB Control: Surveillance, Planning, Financing 2007 Geneva: WHO

[B9] Jogjakarta provincial health office 2005 TB Control Program: Annual Report 2006 Jogjakarta, Jogjakarta provincial health office

[B10] Auer C Sarol J Jr Tanner M Weiss M Health seeking and perceived causes of tuberculosis among patients in Manila, Philippines Trop Med Int Health 2000 5 648 656 10.1046/j.1365-3156.2000.00615.x 11044280

[B11] Portero NJ Rubio YM Pasicatan MA Socio-economic determinants of knowledge and attitudes about tuberculosis among the general population of Metro Manila, Philippines Int J Tuberc Lung Dis 2002 6 301 306 11936738

[B12] Godfrey-Faussett P Kaunda H Kamanga J van Beers S van Cleeff M Kumwenda-Phiri R Tihont V Why do patients with a cough delay seeking care at Lusaka urban health centres? A health systems research approach Int J Tuberc Lung Dis 2002 6 796 805 12234135

[B13] Werf MJ Van der Chechulin Y Yegorova OB Marcinuk T Stopolyanskiy A Voloschuk V Zlobinec M Vassall A Veen J Hasker E Turchenko LV Health care seeking behaviour for tuberculosis symptoms in Kiev City, Ukraine Int J Tuberc Lung Dis 2006 10 390 395 16602402

[B14] Huong NT Vree M Duong BD Khanh VT Loan VT Co NV Borgdorff MW Cobelens FG Delays in the diagnosis and treatment of tuberculosis patients in Vietnam: a cross-sectional study BMC Public Health 2007 7 110 17567521 10.1186/1471-2458-7-110 PMC1906755

[B15] Pehme L Rahu K Rahu M Altraja A Factors related to health system delays in the diagnosis of pulmonary tuberculosis in Estonia Int J Tuberc Lung Dis 2007 11 275 281 17352092

[B16] Hurtig AK Pande SB Baral SC Newell J Porter JDH Bam DS Linking private and public sectors in tuberculosis treatment in Kathmandu Valley, Nepal Health Policy Plan 2002 17 78 89 10.1093/heapol/17.1.78 11861589

[B17] Siddiqi K Newell JN Stuyft P Van der Gotuzzo E Torrico F Van Deun A Walley J Improving sputum microscopy services for the diagnosis of tuberculosis in Peru and Bolivia Int J Tuberc Lung Dis 2007 11 665 670 17519099

[B18] Piot MA A Simulation Model of Case Finding and Treatment in Tuberculosis Control Programmes. WHO/TB/Tech.information/67.53 1967 Geneva: WHO

[B19] Keeler E Perkins MD Small P Hanson C Reed S Cunningham J Aledort JE Hillborne L Rafael ME Girosi F Dye C Reducing the global burden of tuberculosis: the contribution of improved diagnostics Nature 2006 444 Suppl 1 49 57 10.1038/nature05446 17159894

[B20] Millen SJ Uys PW Hargrove J van Helden PD Williams BG The effect of diagnostic delays on the drop-out rate and the total delay to diagnosis of tuberculosis PLoS ONE 2008 3 e1933 18398459 10.1371/journal.pone.0001933 PMC2276686

[B21] De Vlas SJ Cunningham J Ahmad RA Perkins M Nagelkerke NJD Modeling the tuberculosis diagnostic process to predict the impact of new diagnostic technologies Trop Med Int Health 2007 12 Suppl 1 123

[B22] Bowles N The Delphi technique Nurs Stand 1999 13 32 36 10633705 10.7748/ns1999.07.13.45.32.c2650

[B23] Campbell SM Shield T Rogers A Gask L How do stakeholder groups vary in a Delphi technique about primary mental health care and what factors influence their ratings? Qual Saf Health Care 2004 13 428 434 15576704 10.1136/qshc.2003.007815 PMC1743904

[B24] Beattie E Mackway-Jones K A Delphi study to identify performance indicators for emergency medicine Emerg Med J 2004 21 47 50 14734375 10.1136/emj.2003.001123 PMC1756339

[B25] Maiburg BHJ Rethans JJE van Ree JW GPs' needs for practice-oriented nutrition education: a Delphi study among Dutch GPs Fam Pract 2004 21 425 428 10.1093/fampra/cmh413 15249532

[B26] Katcher ML Meister AN Sorkness CA Staresinic AG Pierce SE Goodman BM Peterson NM Hatfield PM Schirmer JA Use of the modified Delphi technique to identify and rate home injury hazard risks and prevention methods for young children Inj Prev 2006 12 189 194 16751451 10.1136/ip.2005.010504 PMC2563522

[B27] WHO An Expanded DOTS Framework for Effective Tuberculosis Control. WHO/CDS/TB/2002.297 2002 Geneva, Switzerland: WHO

[B28] Heller RF Gemmell I Edwards R Buchan I Awasthi S Volmink JA Prioritising between direct observation of therapy and case-finding interventions for tuberculosis: use of population impact measures BMC Med 2006 4 35 17181867 10.1186/1741-7015-4-35 PMC1764027

[B29] Golub JE Mohan CI Comstock GW Chaisson RE Active case finding of tuberculosis: historical perspective and future prospects Int J Tuberc Lung Dis 2005 9 1183 1203 16333924 PMC4472641

[B30] Dye C Floyd F Jamison DT, et al Tuberculosis Disease Control Priorities in Developing Countries 2005 2 New York: Oxford University Press 289 312

[B31] Murray CJL Salomon JA Expanding the WHO tuberculosis control strategy: rethinking the role of active case-finding Int J Tuberc Lung Dis 1998 2 S9 S15 9755959

[B32] Marchand R Tousignant P Chang H Cost-effectiveness of screening compared to case-finding approaches to tuberculosis in long-term care facilities for the elderly Int J Epidemiol 1999 28 563 570 10.1093/ije/28.3.563 10405865

[B33] Zachariah R Spielmann MP Harries AD Gomani P Graham SM Bakali E Humblet P Passive versus active tuberculosis case finding and isoniazid preventive therapy among household contacts in a rural district of Malawi Int J Tuberc Lung Dis 2003 7 1033 1039 14598961

[B34] Noertjojo K Tam CM Chan SL Tan J Chan-Yeung M Contact examination for tuberculosis in Hong Kong is useful Int J Tuberc Lung Dis 2002 6 19 24 11931396

[B35] Becerra MC Pachao-Torreblanca IF Bayona J Celi R Shin SS Kim JY Farmer PE Murray M Expanding tuberculosis case detection by screening household contacts Public Health Rep 2005 120 271 277 16134567 10.1177/003335490512000309 PMC1497729

[B36] Shargie EB Mørkve O Lindtjørn B Tuberculosis case-finding through a village outreach programme in a rural setting in southern Ethiopia: community randomized trial Bull World Health Organ 2006 84 112 119 16501728 10.2471/BLT.05.024489 PMC2626531

[B37] Hamid Salim MA Uplekar M Daru P Aung M Declerq E Lönnroth K Turning liabilities into resources: informal village doctors and tuberculosis control in Bangladesh Bull World Health Organ 2006 84 479 484 16799732 10.2471/BLT.05.023929 PMC2627374

[B38] Floyd K Arora VK Murthy KJR Lonnroth K Singla N Akbar Y Zignol M Uplekar M Cost and cost-effectiveness of PPM-DOTS for tuberculosis control: evidence from India Bull World Health Organ 2006 84 437 445 16799727 10.2471/BLT.05.024109 PMC2627367

[B39] Borgdorff MW Floyd K Broekmans JF Interventions to reduce tuberculosis mortality and transmission in low- and middle-income countries Bull World Health Organ 2002 80 217 227 11984608 PMC2567749

[B40] Macintyre CR Plant AJ Hendrie D The cost-effectiveness of evidence-based guidelines and practice for screening and prevention of tuberculosis Health Econ 2000 9 411 421 10.1002/1099-1050(200007)9:5<411::AID-HEC524>3.0.CO;2-9 10903541

[B41] Dasgupta K Schwartzman K Marchand R Tennenbaum TN Brassard P Menzies D Comparison of cost-effectiveness of tuberculosis screening of close contacts and foreign-born populations Am J Respir Crit Care Med 2000 162 2079 2086 11112118 10.1164/ajrccm.162.6.2001111

[B42] Wyss K Kilima P Lorenz N Cost of tuberculosis household and health care providers in Dar es Salaam, Tanzania Trop Med Int Health 2001 6 60 68 10.1046/j.1365-3156.2001.00677.x 11251897

[B43] Ministry of Health of Indonesia Report of the Joint External TB Monitoring Mission Indonesia (16–27 April 2007) 2007 Jakarta: Ministry of Health of Indonesia

